# Sensory Perception of Food and Insulin-Like Signals Influence Seizure Susceptibility

**DOI:** 10.1371/journal.pgen.1000117

**Published:** 2008-07-04

**Authors:** Todd R. Gruninger, Daisy G. Gualberto, L. Rene Garcia

**Affiliations:** Department of Biology, Texas A&M University, College Station, Texas, United States of America; Stanford University Medical Center, United States of America

## Abstract

Food deprivation is known to affect physiology and behavior. Changes that occur could be the result of the organism's monitoring of internal and external nutrient availability. In *C. elegans*, male mating is dependent on food availability; food-deprived males mate with lower efficiency compared to their well-fed counterparts, suggesting that the mating circuit is repressed in low-food environments. This behavioral response could be mediated by sensory neurons exposed to the environment or by internal metabolic cues. We demonstrated that food-deprivation negatively regulates sex-muscle excitability through the activity of chemosensory neurons and insulin-like signaling. Specifically, we found that the repressive effects of food deprivation on the mating circuit can be partially blocked by placing males on inedible food, *E. coli* that can be sensed but not eaten. We determined that the olfactory AWC neurons actively suppress sex-muscle excitability in response to food deprivation. In addition, we demonstrated that loss of insulin-like receptor (DAF-2) signaling in the sex muscles blocks the ability of food deprivation to suppress the mating circuit. During low-food conditions, we propose that increased activity by specific olfactory neurons (AWCs) leads to the release of neuroendocrine signals, including insulin-like ligands. Insulin-like receptor signaling in the sex muscles then reduces cell excitability via activation of downstream molecules, including PLC-γ and CaMKII.

## Author Summary

We demonstrate that lack of sensation of food in the environment can alleviate spontaneous muscle seizures via an insulin-like mediated pathway. Food restriction is known to promote many adaptive physiological responses, including the mobilization of fat-stores, increases in life span, and suppression of seizures. Consequently, a better understanding of the exact cellular and molecular details involved in these responses could lead to better health treatments. Here, we identify a novel mechanism activated by food restriction that occurs as a response to decreased sensation of food in the environment, rather than a response to decreased ingestion. Specifically, we show that suppression of *C. elegans* mutant-induced muscle seizures by food deprivation requires the activity of the AWC olfactory neurons and insulin-like signaling. We elucidate a novel role of insulin-like signaling in response to caloric restriction that does not involve the canonical FOXO/DAF-16 pathway. Instead, we propose that insulin-like control of muscle excitability activates downstream signaling molecules that include phospholipase C-γ, calcium/calmodulin-dependent kinase II (CaMKII), and *ether-a-go-go* K^+^ channels. These molecules identify a novel mechanism used by organisms to regulate multiple physiological responses to changing environmental conditions.

## Introduction

The feeding status of an organism can alter physiology and motor output leading to changes in health and behavior. For example, food deprivation can improve stress resistance, increase life-span, and alleviate muscle seizures [1]–[7]. In addition to promoting these phenomena under food-deprived conditions, organisms must also attenuate circuits not involved in food-foraging, such as reproductive behaviors. Although food deprivation has been established to modulate the physiology of multiple neuromuscular circuits in different species, the detailed mechanisms that integrate these circuits are just beginning to emerge. Since an organism's experience of food consists of multiple sensory cues such as odor, texture, and temperature, it is likely that physiological responses to food are dependent on both sensory perception and ingestion of food. Here, we use the regulation of *C. elegans* male sex-muscle excitability under well-fed and food-deprived conditions to dissect these mechanisms.

In the laboratory, *C. elegans* male mating behavior normally occurs on a food source (*E. coli* OP50) when a well-nourished male encounters a hermaphrodite. Contact with a hermaphrodite by sensilla in the male tail causes the male to stop forward locomotion, and begin scanning the hermaphrodite for its vulva. Once the male has located the vulva, he rapidly contracts sex muscles, which consist of two retractor muscles and two protractor muscles attached to each of his two copulatory spicules. Once his spicules breach the vulva, he maintains a tonic contraction of his sex muscles to keep the spicules inserted while sperm is transferred [1], [8]–[13]. In contrast to food-satiated males, we find that food-deprived males mate with less efficiency, suggesting that the excitability of the muscles and neurons controlling mating is reduced.

Loss-of-function mutations in the gene *unc-103*, which encodes an ERG-like K^+^ channel, cause well-fed males to display spontaneous spicule-muscle seizures [14]. Interestingly, food-deprivation suppresses the seizures of *unc-103(lf)* mutants. We have previously demonstrated that suppression of *unc-103*-induced muscle seizures requires calcium/calmodulin-dependent kinase II (CaMKII) and *ether-a-go-go* (EAG) -like K^+^ channel activity in the sex muscles [1]. This pathway could be activated by a sensory response to decreased food in the environment or an internal response to decreased ingestion of food.

In this study, we investigate mechanisms that act upstream of the sex-muscle CaMKII/EAG K^+^ channel pathway that reduce seizure susceptibility under food-deprived conditions. Our results demonstrate that sensation of food, independent of ingestion, can influence muscle excitability. We propose that under low-food conditions, AWC olfactory neuron activity signals to the sex muscles to suppress cell excitability via the insulin-like receptor, DAF-2. Additionally, we find that DAF-2 does not regulate sex-muscle excitability by signaling through the canonical FOXO/DAF-16 transcription factor. Instead, our observations suggest that DAF-2 signals to CAMKII via the *C. elegans* phospholipase C-γ (PLC- γ), PLC-3, to suppress seizures.

## Results

### Male Mating Behaviors Are Suppressed by Food Deprivation

Male mating behavior initiates when sensilla in the male tail contact a hermaphrodite. The male then presses the ventral portion of his tail against the hermaphrodite's cuticle and begins moving backwards, keeping tail contact with the hermaphrodite and scanning for her vulva. If the male reaches the end of the hermaphrodite without sensing the vulva, he performs a ventral turn and scans the other side. Once he locates the vulva, he halts backward locomotion and attempts to insert his copulatory spicules into his mate. Spicule insertion is achieved by rapid contraction of his sex muscles, which consist of two retractor muscles and two protractor muscles attached to each of his two spicules. Once he has breached the vulva, he maintains a tonic contraction of his sex muscles, which allows sperm to be transferred into his mate [8]–[12].

**Figure 1:**
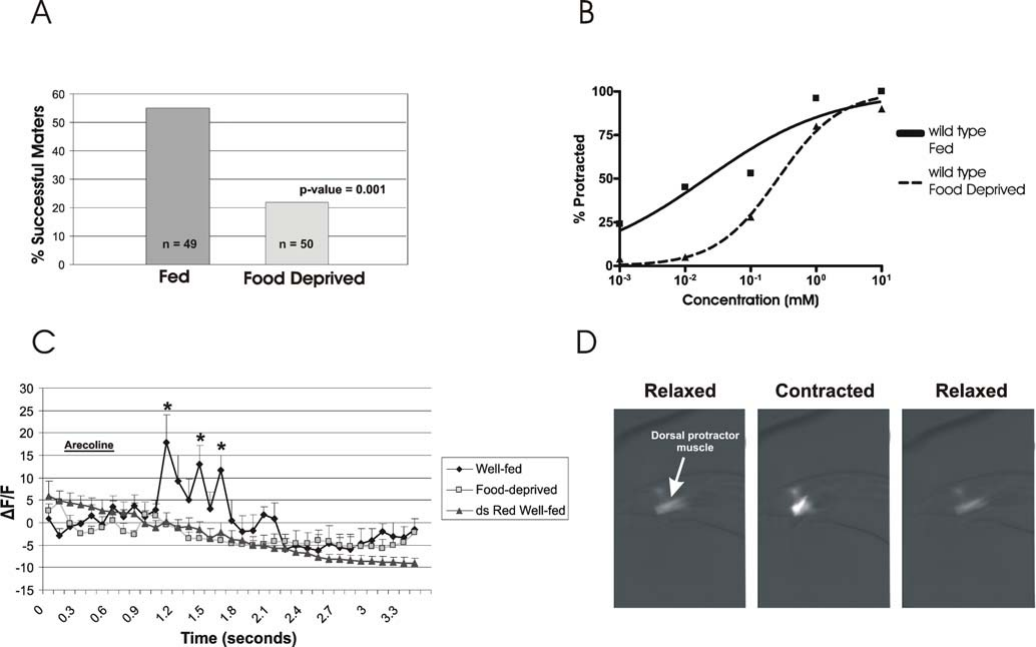
Effects of food deprivation on male sex-muscle excitability. (A) Mating success for fed and 15hr food-deprived males. Males were scored as successful if they sired at least 1 progeny. p-value determined by Fisher's exact test (B) Graph of male muscle arecoline (ARE) sensitivity. For each concentration assayed, 20–30 males were assayed. (C) Mean sex-muscle G-CaMP responses to 10mM ARE for fed (n = 4) and food-deprived (n = 4) males. The * denotes well-fed time points that are significantly different (p≤0.05, Bonferroni posttest) then the food-deprived control. For fed males, the mean dsRed intensity trace is shown as a control. Error bars represent standard error of the mean. (D) Three representative frames displaying ARE-induced calcium changes in the sex muscles of a well-fed male (time between each frame is approximately 0.7 seconds). Anterior is to the right and the dorsal protractor muscles are labeled. Scale bar 6 µm.

We have found that food-deprived males are less effective at mating behavior than well-fed males. To quantify this observation, we tested the effects of food deprivation on wild-type mating success (Figure 1A). We paired both well-fed males and 15-hr food-deprived males with hermaphrodites and scored the number of males that could sire at least one progeny given a 2-hr mating interval. About 55% of well-fed males could sire at least one progeny, while only 20% of food-deprived males were successful (p = 0.001, Fisher's exact test). The reduced mating success of food-deprived males could be due to multiple factors. Since mating behavior consists of multiple sub-steps, we focused on the spicule insertion step of mating, as inefficacy at this step is a likely reason for unsuccessful mating.

Food deprivation can suppress precocious spicule protraction caused by the loss of the ERG-like K^+^ channel, UNC-103 (Table 1). We have previously reported that UNC-103 acts in the sex muscles to suppress muscle excitability and spontaneous spicule protraction. We reasoned that in *unc-103* deletion (*unc-103(0)*) males, food-deprivation could compensate for loss of the gene by activating other mechanisms that reduce muscle output [1],[13]. Therefore, it is possible that food deprivation reduces mating efficiency of wild-type males by suppressing pathways that increase sex-muscle excitability. Previous genetic analyses on the male circuitry have revealed that spicule muscle behaviors are regulated by at least two distinct signaling pathways [12]. Specifically, the neurons controlling sex-muscle output activate different spicule muscle ACh receptors and downstream signaling pathways to mediate distinct muscle contractile behaviors. The ACh agonist levamisole activates spicule protraction through the ryanodine receptor (RyR), UNC-68, while the ACh agonist arecoline activates spicule protraction through the L-type voltage-gated Ca^2+^ channel (L-VGCC), EGL-19 [12]. Other tissues and physiological responses (i.e. food availability) integrated with male mating may act on one or both of these pathways. We have recently demonstrated that food deprivation reduces *unc-103(0)*-mediated spicule protraction by affecting a pathway that includes CaMKII and L-VGCC/EGL-19 channels [1], suggesting that reduced mating efficiency in food-deprived males may be due to suppression of L-VGCC/EGL-19 activity. To verify in wild-type males that food deprivation results in a reduction of L-VGCC/EGL-19-induced Ca^2+^ influx and muscle contraction, we tested sex-muscle response to arecoline [12]. Similar to mating success, we found that food-deprivation reduces wild-type response to arecoline-induced spicule protraction (Figure 1B). The effective concentration at which 50% of well-fed males protracted their spicules (EC50) was 21 µM while food-deprived males had an EC50 of 262 µM.

**Table 1:** Chemosensory mutations block food-deprivation suppression of sex-muscle excitability.

Genotype	% Protracted on Food	% Protracted on Inedible Food	% Protracted on No Food
*unc-103(0)*	39 (102)^a^	20 (85)	9 (101)
*osm-5(p813)*	0 (10)	0 (10)	0 (10)
*osm-9(ky10)*	6 (32)	5 (20)	9 (34)
*unc-103(0) osm-5(p813)*	33 (163)	22 (51)	20^b^ (96)
*unc-103(0); osm-9(ky10)*	41 (59)	28 (40)	21^b^ (56)

a Numbers in parentheses refer to the number of animals assayed.

b Significantly different than control No Food condition p<0.01, Fisher's Exact Test.

In addition to measuring spicule protraction, we used the fluorescent calcium indicator, G-CaMP [15], to measure real-time calcium changes in the sex muscles of fed and food-deprived males (Figure 1C-D). G-CaMP consists of a circularly permutated enhanced GFP connected to the M13 fragment of myosin light chain kinase and calmodulin (CaM). When Ca^2+^ binds to the CaM of G-CaMP, CaM binds to the M13 region and the induced conformational changes result in fluorescent intensity changes [15]. We hypothesized that reduced response to ACh agonist in starved-males was due to decreased calcium signaling in the spicule muscles. To drive expression of G-CaMP in the sex muscles, we used the *unc-103 1E* promoter, which expresses in the spicule protractor muscles [16]. Additionally, we also co-injected dsRed as a control for intensity changes caused by reduced cell volume or unintentional background changes. As expected, we found that spicule protractor muscles of food-deprived males had significantly lower calcium responses to arecoline (Figure 1C). Figure 1C displays the averaged G-CaMP and dsRed trace for well-fed (n = 4) and the averaged G-CaMP trace for 2hr food-deprived (n = 4) males. Specifically, starved males did not show an increase in intensity in response to arecoline, while fed males showed rhythmic increases in intensity over time (Figure 1C). These observations suggest that active mechanisms in food-deprived males reduce calcium signaling and muscle contraction in response to stimulation. Taken together with previous results, we suggest that food deprivation suppresses sex-muscle excitability of both wild-type and *unc-103(0)* males, and the phenotype of *unc-103(0)* males can be used to track how different perturbations affect this regulation.

### Food Deprivation Suppresses Sex-Muscle Excitability via Sensory Signals

**Figure 2:**
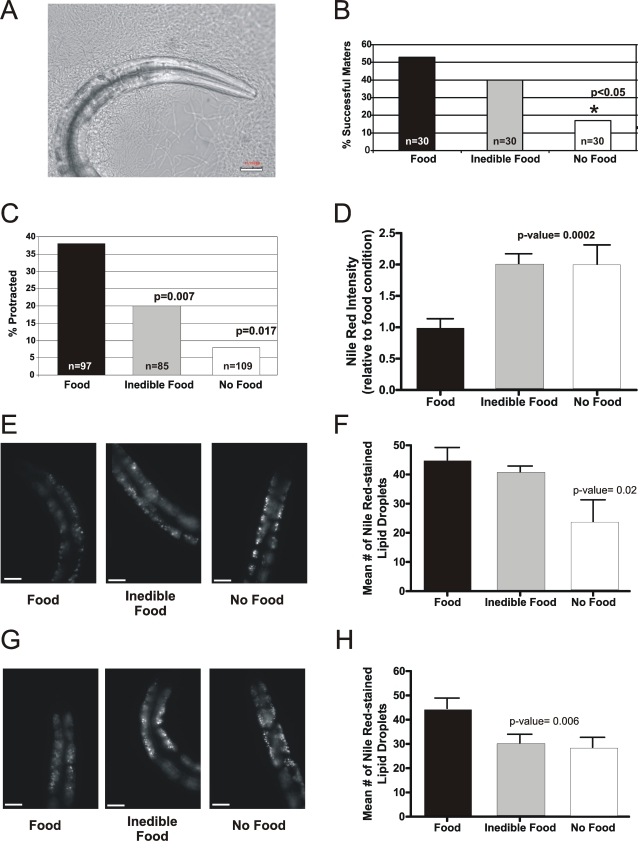
Food deprivation suppresses sex-muscle excitability via both internal and external sensory responses. (A) Adult male on aztreonam-treated *E.coli* OP50. Scale bar 20 µm. (B) Effects of food, inedible food, and no food on wild-type mating efficiency. p-value listed using Fisher's exact test. (C) Effects of food, inedible food, and no food on *unc-103(0)*-induced seizures. p-value listed using Fisher's exact test. (D) Bar graph representing the effects of food, inedible food and no food on Nile Red fluorescent intensity for wild-type males (E) Representative Nile Red-stained wild-type males under the three different feeding conditions (scale bar and 23.5 µm). (F) Bar graph representing the effects of different feeding conditions on the mean number of Nile Red-stained fat droplets for wild-type males. (G) Representative Nile Red-stained *Posm-12:unc-103(gf)* males under the three different feeding conditions (scale bar and 23.5 µm). (H) Bar graph representing the effects of different feeding conditions on the mean number of Nile Red-stained fat droplets in *Posm-12:inc-103(gf)* males. For each genotype and condition, 10 males were analyzed for Nile Red staining and the p-values were determined using the Student's t test.

We previously identified CaMKII (UNC-43) and an EAG K^+^ Channel (EGL-2) as components of a signaling pathway that reduces excitability under food-deprived conditions. However, the upstream signals that activate this pathway under starved conditions were not clear [1]. In food-deprived males, reduced sex-muscle excitability could be the result of two possibilities: as a response to decreased ingestion and nourishment from food, and/or a response to decreased sensation of food in the environment. To differentiate between these two hypotheses, we asked if exposing males to inedible *E. coli*, i.e. bacteria they can sense but not ingest, is similar to depriving them of food. We treated *E. coli* OP50 with the antibiotic azetreonam, which inhibits cell wall septation [17], resulting in long chains of bacteria (5 to >50 µm) that are too long to fit inside *C. elegans'* mouth (Figure 2A). To observe effects on wild-type mating efficiency, we separated L4 larval-stage males and placed them on one of three conditions: standard growth plates with food (untreated *E. coli*), with inedible food (*E. coli* treated with azetreonam), and with no food (no bacteria present). Interestingly, we found that wild-type males placed on inedible bacteria for 15hrs prior to the mating efficiency were more likely to successfully mate than their food-deprived counterparts (Figure 2B).

In addition to measuring mating efficiency, we also measured the effects of inedible bacteria on *unc-103(0)*-induced seizures. The *unc-103(0)* deletion allele caused 38% of males to display spontaneous spicule-muscle seizures on food, whereas only 8% of males placed on the no-food condition displayed the defect. Interestingly, 20% of *unc-103(0)* males placed on plates with inedible bacteria had spontaneous muscle seizures, which is significantly different from both food (p = 0.007) and no-food (p = 0.017) conditions (Figure 2C and Table 1). Taken together, these results suggest that both sensation of food and ingestion of food can influence male mating behavior, possibly through regulating the excitability of the sex muscles.

Although our data indicates that inedible bacteria can partially block the effects of food deprivation, it is possible that males could be breaking up and ingesting some bacteria, albeit at a reduced amount. However, males placed with no *E. coli* or aztreonam-treated *E. coli* shared similar phenotypes, including a pale appearance and empty intestines. To rule out that aztreonam-treated *E coli* are edible, we used Nile Red to visualize if internal fat stores are utilized in the three different conditions [18]. Males were grown on plates with Nile Red until L4-larval stage and then moved to one of three different feeding plates: with *E. coli*, inedible *E. coli*, or no *E. coli*. We then used two different standards of measurement to compare fat staining between males grown in different conditions: fluorescent intensity and the number of Nile Red-stained fat droplets. Interestingly, we found that both food-deprived males and males placed on inedible bacteria had significant increases in Nile Red intensity staining compared to well-fed males (Figure 2D-E). This result suggests that the fat regulatory mechanisms activated in food-deprived males are similarly activated in males on inedible bacteria. However, in contrast to fat droplet intensity, we found that males grown on aztreonam-treated and non-treated bacteria had significantly higher numbers of Nile Red-stained fat droplets than males grown without bacteria (Figure 2F). Therefore, although males grown on aztreonam-treated bacteria showed similar overall increases in fat-droplet intensity, our data suggests that males grown on aztreonam-treated bacteria are not mobilizing fat stores as quickly as food-deprived males.

To reconcile the fact that males placed on azrtreonam-treated *E. coli* looked similar to starved males yet had larger numbers of fat stores, we hypothesized that sensation of bacteria could partially block fat mobilization. To test this, we transgenetically expressed a mutant gain-of-function UNC-103 K^+^ channel in chemosensory neurons using the *osm-12* promoter [19],[20], and measured effects on the number of Nile Red stained foci (Figure 2G-H). The transgenic UNC-103(gf) K^+^ channel contains an A331T change in the sixth transmembrane spanning domain (S6) [16], [21]–[23]. This *Posm-12:unc-103(gf)* construct should hyperpolarize chemosensory neurons, reducing their ability to transduce olfactory signals. To confirm the construct reduces the function of chemosensory neurons, we verified that the animals showed previously identified chemotaxis defects (Figure S1A) [24],[25]. If chemosensation of bacteria blocks fat mobilization, males expressing *Posm-12:unc-103(gf)* should show a decrease in fat stores on aztreonam-treated bacteria. This was indeed the case; *Posm-12:unc-103(gf)* males had significantly lower numbers of fat droplets on both aztreonam-treated bacteria and on no food (Figure 2H). Although the increased Nile Red intensity and differences in the number of stained foci raise interesting questions about fat homeostasis that are beyond the scope of this work, our results suggest that males placed on aztreonam-treated bacteria are similarly food-deprived as males cultured with no bacteria.

In addition to visualizing fat storage in males placed on inedible bacteria, we used GFP-expressing bacteria to visualize edibility, and also tested the ability of aztreonam-treated bacteria to sustain developmental growth. As expected, we found that untreated GFP-expressing *E. coli* could be visualized in the isthmus of the pharynx, the grinder, and in the intestines (Figure S1B-C). Although the worms grinded up most of the bacteria before passage to the intestines, for 95% (n = 20) of males observed, we detected small amounts of intact GFP-expressing bacteria in the intestine (n = 20). In contrast, when we treated the GFP-expressing bacteria with aztreonam, we never detected any fluorescent *E. coli* in the intestines (n = 20) (Figure S1C). In 55% of the males placed on inedible bacteria, single, long chains of bacteria were seen trapped in the isthmus of the pharynx, suggesting that few of the aztreonam-treated bacteria are small enough to enter the pharynx, but too large to pass through the grinder and intestines. To verify that aztreonam-treated *E. coli* are not digested and cannot sustain growth, we placed starved L1 larva on the three different feeding plates and visualized growth rate during our standard 15hr period. *C. elegans* hatch in the developmentally arrested L1 stage and only initiate postembryonic development in the presence of food (bacteria) [26]–[28]. Thus, we predicted that worms placed on aztreonam-treated *E. coli*, should arrest at L1, similar to worms placed on plates with no food. We monitored the number of gonadal cell nuclei to compare the relative developmental stage of worms grown under the three different feeding conditions (Figure S1D). We found that synchronized L1s that were placed on food for 15hrs all contained 12-celled gonads. In contrast, synchronized L1s that were placed on either aztreonam-treated bacteria or no bacteria for 15hrs were arrested, containing 4-celled gonads. Similar to worms grown without bacteria, our results suggest that worms grown on aztreonam-treated bacteria are not ingesting and breaking down nutrients required for developmental growth. Therefore, we conclude that aztreonam-treated bacteria are inedible, and the sensation of the inedible bacteria can block various food-deprivation physiological responses, including mobilization of fat stores and reduced muscle excitability.

### Chemosensory Neurons in the Head Regulate Sex-Muscle Excitability

*C. elegans* uses chemosensation to locate the source of attractive odors (presumably for food), to avoid noxious odors, and to determine whether to enter the dauer diapause stage [29]–[31]. In addition, males use chemosensation to locate and mate with hermaphrodites [32]–[34]. To accomplish these tasks, worms use chemosensory neurons that have sensory cilia exposed to the environment, which can react to various chemicals. In regard to male mating, our finding that inedible food can partially block the suppressive effects of food deprivation suggests that chemosensory neurons down-regulate sex-muscle excitability, and their activity is attenuated when food stimulus is present. To determine if lack of sensing *E. coli* partially suppresses sex-muscle excitability, we assayed known chemosensory mutants. We generated double-mutants with *unc-103(0)* and *osm-5(p813)* and *osm-9(ky10)*, and assayed the effects on muscle seizures in the three different feeding conditions (Table 1). Neither chemosensory mutation significantly affected the percentage of protracted males under the inedible-food condition; however, each mutation significantly increased the percentage under the no-food condition. These results are consistent with chemosensory neurons actively suppressing sex-muscle excitability under food-deprived conditions, and chemosensation of bacteria repressing the neurons' activity.

To determine which chemosensory neurons regulate spicule protraction in response to food-status, we transgenetically expressed the UNC-103(gf) K^+^ channel in specific groups of chemosensory neurons. We first used the general chemosensory *osm-12* promoter to see if it phenocopied the effects of the chemosensory mutations. We did not use the promoters for *osm-5* or *osm-9*, since their full expression pattern requires coding sequences that could potentially interfere with the UNC-103 K^+^ channel fusion. When we expressed *unc-103(gf)* in all chemosensory neurons of *unc-103(0)* males, we found that *unc-103(0)*-induced seizures were no longer suppressed by food deprivation (Table 2). These results are consistent with the ability of *osm-5* and *osm-9* mutations to block food-deprivation suppression of seizures.

**Table 2:** Chemosensory neurons suppress *unc-103(0)*-seizures in the absence of food stimulus.

Transgene	Cells Affected	% Protracted on Food	% Protracted on Inedible Food	% Protracted on No Food
None^a^	None	30 (56)^b^	15 (34)	3 (63)
*rgEx208* [*Posm-12:unc-103(gf)*]	All ciliated neurons	33 (55)	21 (34)	18^c^ (89)
*rgEx234* [*Ptax-2:unc-103(gf)*]	AFD, AQR, ASE, ASI, AWC, BAG	36 (89)	24 (41)	21^c^ (56)
*rgEx205* [*Pocr-2:unc-103(gf)*]	ADF, ADL, ASH,AWA, PHA, PHB	38 (47)	23 (30)	9 (32)
*rgEx201* [*Podr-3:unc-103(gf)*]	AWA, AWB, AWC	35 (37)	22 (36)	23^c^ (43)

a All genotypes contain *unc-103(0).*

b Numbers in parentheses refer to the number of animals assayed.

c Significantly different than the non-transgenic no-food condition p<0.01, Fisher's Exact Test.

**Figure 3:**
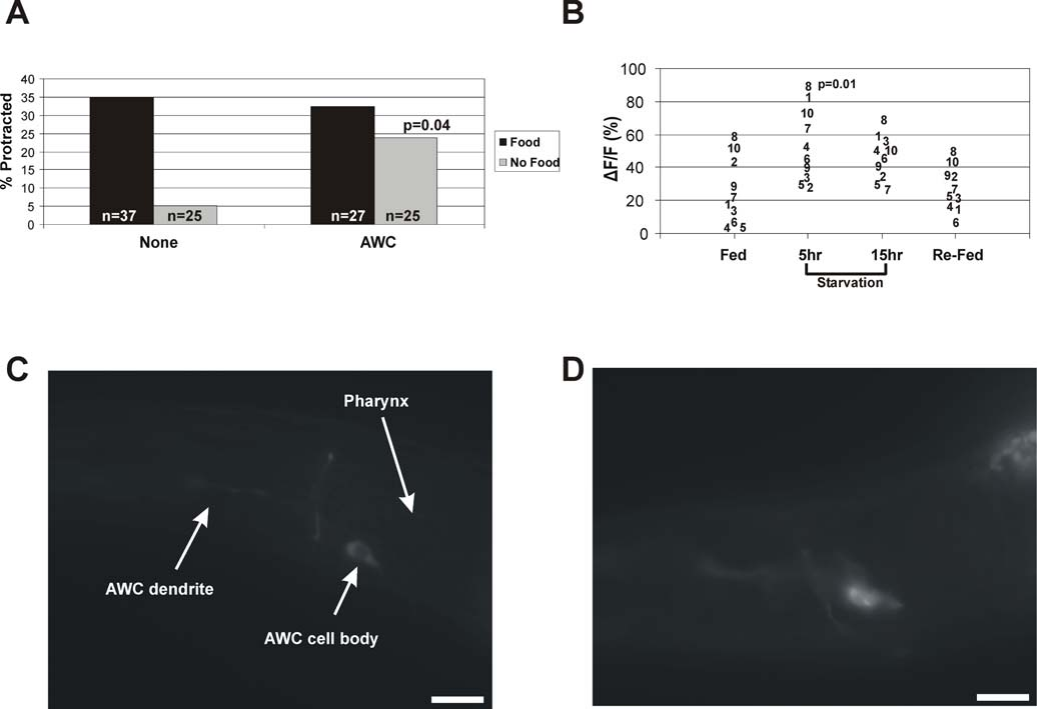
The olfactory AWC neurons regulate sex-muscle excitability in response to environmental conditions. (A) Graph showing the effect of laser ablation of the AWC olfactory neurons. The * denotes a significant difference (Fisher's exact test) compared to mock ablated no-food control. (B) G-CaMP intensity changes in the left AWC neurons of 10 individual males taken after fed, 5hr and 15hr of food deprivation, and re-fed. Each number (1–10) identifies the same male for each interval. The p-value listed signifies a significant difference between Fed and 5-hr food-deprived males. (C) A representative G-CaMP intensity image of a well-fed and (D) 15hr food-deprived male (scale bar 9 µm).

Since our transgenic gain-of-function K^+^ channel reduced chemosensory function, we then expressed it using more restrictive promoters (Table 2). The promoters and their expression pattern confirmed by YFP reporters were as follows: *Ptax-2* (AFD, AQR, ASE, ASI, ASK, AWC, BAG), *Pocr-2* (ADF, ADL, ASH, AWA, PHA, PHB), and *Podr-3* (AWA, AWB, AWC, PHA, PHB) [35]–[37]. Reducing the excitability of *ocr-2*-expressing neurons did not affect food-deprivation suppression of sex-muscle seizures, suggesting that the ADF, ADL, ASH, AWA, PHA, and PHB neurons are not involved. In contrast, reducing the function of either *tax-2* or *odr-3*-expressing neurons significantly increased the percentage of starved males that had spontaneous seizures (Table 2). Since only the AWC neurons express both *tax-2* and *odr-3*, it is likely that these neurons suppress sex-muscle excitability under food-deprived conditions. We therefore tested if the AWCs are required for food-deprivation suppression of mating behavior via *Podr-3:unc-103(gf)* and through cell-ablation (Figure S1E). However, instead of increasing mating potency under food-deprived conditions, we found that removing the AWCs significantly decreased mating efficiency in well-fed males. During the assays, we noticed that AWC-ablated (genetically or with laser) males rarely encountered the hermaphrodite during the 2hr mating interval (data not shown). This finding is similar to the observations of White and colleagues, which demonstrated that the AWCs are required for male chemotaxis to hermaphrodite pheromones [32]. Therefore, it is likely that AWC functions in multiple aspects of male behavior, and its role in chemotaxis to hermaphrodites could explain why AWC-ablated males have reduced mating efficiency. Additionally, there may be other redundant neurons that can suppress mating efficiency in response to food deprivation. To test the role of the AWCs on sex-muscle excitability more directly, we ablated the neurons in *unc-103(0)* males and assayed the number of fed and food-deprived males with seized muscles. Similar to the *Podr-3:unc-103(gf)* and *Ptax-2:unc-103(gf)* experiments, AWC-ablated *unc-103(0)* males had a significantly higher probability of having sex-muscle seizures under food-deprived conditions than the mock-ablated controls (Figure 3A). Although we cannot conclude that the AWCs are sufficient or rule out potential roles for other neurons, our data indicates that the AWC neurons regulate the male genitalia in response to food availability.

Since the AWCs are required to suppress sex-muscle excitability under starved conditions, these neurons may be more active when no food is being sensed in the environment, and attenuated while food is present. Consistent with this observation, Chalasani et al [38] used the fluorescent calcium indicator G-CaMP [15] to demonstrate that the AWC neurons are activated by the removal of odorants such as isoamyl alcohol and benzaldehyde, and remain active in the absence of these chemicals. To verify that AWC responses to odourant presence and removal corresponded to the presence or absence of food in our assays, we used G-CaMP to measure the calcium activity of the AWC neurons during fed, food-deprived, and re-fed conditions (Figure 3B-D). We transgenetically expressed G-CaMP from the *odr-3* promoter and used 10 well-fed adult males to measure G-CaMP fluorescence intensity after 5 hours of food deprivation, 15 hours of food deprivation, and after 5hr re-feeding. Consistent with the findings of Chalasani and co-workers [38], we found that the AWC neurons significantly increase in fluorescence intensity during the 5 and 15 hour starvation period, and then decrease back to original levels when the male is re-fed (Figure 3B-D). These results suggest that the AWC olfactory neurons are initiating signals under food-deprived conditions that lead to decreased sex-muscle excitability. The mechanism for how the AWCs communicate to the sex muscles is not clear; it is possible that they signal through cell-cell communication or through regulating the secretion of neuroactive peptides.

### Food Deprivation Requires the Insulin-Like Receptor, DAF-2

In *C. elegans*, one of the most well-studied neuropeptide receptors is the insulin-like receptor, DAF-2. Historically, DAF-2 has been well characterized for its role in regulating physiological responses to food availability, including dauer formation, life-span, and fat homeostasis [39]–[43]. More recently, there is growing evidence that insulin-like signaling also appears to regulate behavioral responses to food, such as male mate searching behavior [33], thermotaxis to temperatures previously associated with food [44], and associating salt with food-deprived conditions [45]. Therefore, we hypothesized that one mechanism for AWC regulation of sex-muscle excitability could be through regulating the secretion of insulin-like peptides.

**Figure 4:**
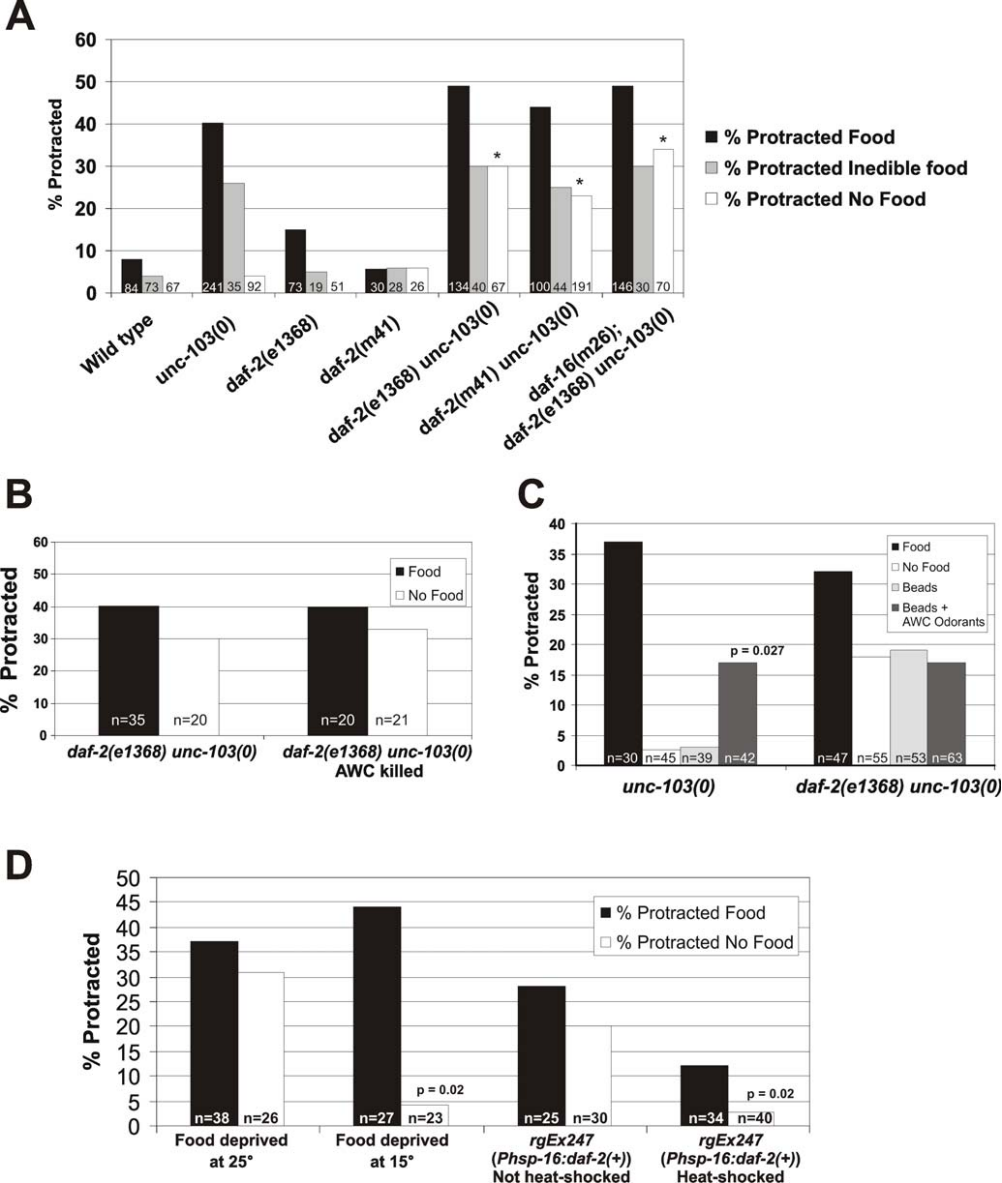
The DAF-2/Insulin-like receptor suppresses sex-muscle excitability under food-deprived conditions. (A) Graph showing the effects of *daf-2(lf)* mutations on *unc-103(0)*-seizure susceptibility in food, inedible food, and no food conditions. The * indicates that the p-value is significantly different (p<0.05) compared to the *unc-103(0)* control condition (Fisher's exact test). (B) Graph displaying the effect of ablating the AWC neurons in *daf-2(e1368) unc-103(0)* mutant males (p-value Fisher's exact test). (C) Graph displaying the effect of AWC odorants (10^−4^ isoamyl alcohol, 10^−7^ 2,3-pentanedione, and 10^−4^ benzaldehyde) on *unc-103(0)*-induced seizures in *unc-103(0)* and *daf-2(e1368) unc-103(0)* males. Sephadex G-50 beads were used as a vehicle for the odorants (p-value Fisher's exact test). (D) Temperature shift assay and heat-shock rescue for *daf-2(e1368ts) unc-103(0)*. Animals were grown at the restrictive temperature (25°C) and then food-deprived at either the restrictive or permissive temperature (15°C). For heat-shock rescue, L4 males were heat-shocked for 35 minutes prior to the assay. The p-values listed were calculated using Fisher's exact test.

To test if the insulin-like pathway is used in food-deprivation induced suppression of seizures, we looked at two temperature sensitive *daf-2* alleles. Interestingly, similar to the chemosensory mutants, we found that *daf-2(e1368)* and *daf-2(m41)* mutants significantly increased *unc-103(0)*-induced seizures under the no-food condition, while having no significant effect on the food or inedible-food conditions (Figure 4A). These results suggest that the insulin-like receptor, DAF-2, is required to suppress sex-muscle excitability under food-deprived conditions. Additionally, neither *daf-2* allele significantly affected the percentage of protracted males on inedible-food. Inedible food is likely suppressing via a non-sensory component, and our data suggests that this component is still intact in *daf-2(ts)* mutants; instead, DAF-2 likely acts downstream of sensory neurons.

Since our results indicate a role for the AWC chemosensory neurons in food-deprivation suppression of sex-muscle output, we hypothesize that the AWC's likely mediate their effects through controlling release of insulin-like peptides. To test if the AWCs signal through DAF-2, we performed the AWC ablation in *daf-2(e1368) unc-103(0)* double mutants. If the AWC's act through insulin-like signaling to suppress sex-muscle excitability, then we predicted that the AWC ablation should not affect the percentage of food or food-deprived *daf-2(e1368) unc-103(0)* males with seized muscles. However, if they function via separate mechanisms, we predicted that ablation of AWC in the double mutant background should significantly increase the percentage of food-deprived males with protracted spicules. Interestingly, we found that ablation of the AWC neurons in *daf-2(e1368) unc-103(0)* males did not significantly affect the percentage of males with seized muscles on either condition assayed (Figure 4B). Additionally, to mimic the inedible food condition which blocks AWC-mediated suppression of sex-muscle excitability, we soaked Sephadex beads (20–80 µM) in odorants previously shown to reduce AWC activity, isoamyl alcohol, benzaldehyde, and 2, 3-pentanedione [38]. We found that beads soaked with AWC-inhibiting odorants increased the percentage of food-deprived *unc-103(0)* males with protracted spicules, but had no significant effect on *daf-2(e1368) unc-103(0)* males (Figure 4C). Taken together, these results suggest that DAF-2 acts downstream of the AWC chemosensory neurons to suppress spontaneous sex-muscle output.

DAF-2 could be regulating sex-muscle excitability acutely by acting in excitable cells at the time of food deprivation, or it could be required developmentally to ensure cells are able to respond when the environment changes. To distinguish between these possibilities, we raised *daf-2(e1368) unc-103(0)* males at the restrictive temperature (25°C) during late L2 through L4 larval stages and then food deprived them as late L4/adult molt at either the permissive (15°C) or restrictive temperature (25°C). If the receptor is required developmentally, then raising the *daf-2(ts)* mutants at 25°C should block food-deprivation suppression, regardless of the temperature at which they are food-deprived. On the contrary, if DAF-2 is required acutely at the time of food deprivation, then the *daf-2(ts)* mutations should not block suppression of seizures when food deprived at 15°C. We found that *daf-2(e1368) unc-103(0)* males raised at 25°C and then food-deprived at 15°C behaved like *unc-103(0)* single mutants; i.e. they were significantly suppressed for spontaneous muscle seizures (Figure 4D). These results suggest that DAF-2 activity is required acutely to suppress sex-muscle excitability. However, it is possible that *daf-2(e1368)* is not truly wild-type at 15°C, and the effects observed may be due to temperature changes rather than true wild-type function of *daf-2*. To rule out this possibility, we rescued *daf-2(e1368)* in *daf-2(e1368) unc-103(0)* mutants using the inducible heat-shock promoter, *Phsp-16* (Figure 4D). Similar to the temperature shift assay, we found that L4 stage males heat-shocked 30min prior to our 15hr food deprivation assay had a significantly lower probability of spontaneous sex-muscle seizures. Additionally, although not quite statistically significant, the *Phsp-16:daf-2(+)* construct appeared to reduce spontaneous spicule protraction in well-fed males as well, suggesting that over-expression of *daf-2* may be able to compensate for loss of *unc-103* function.

In *C. elegans*, many *daf-2(lf)*-mediated behavioral, metabolic and developmental responses require the FOXO transcription factor, DAF-16 [46]–[48]. Previous studies have shown that DAF-2 regulates longevity and developmental pathways by inhibiting DAF-16 from entering the nucleus and activating downstream genes [49]–[52]. To determine if DAF-2 acts on DAF-16 to regulate sex-muscle excitability during food deprived conditions, we constructed the triple mutant, *daf-16(m26); daf-2(e1368) unc-103(0)* and tested the effects of the mutations under the three different food conditions. We found that mutation of *daf-16* did not significantly alter the percentage of males with spontaneous seizures for any of the three-food conditions (Figure 4A). Thus, in contrast to dauer and longevity regulation, DAF-2 is acting through a DAF-16-independent signaling pathway to regulate sex-muscle excitability.

### Sex-Muscle DAF-2 Suppresses Muscle Seizures in Response to Food Deprivation

DAF-2 is broadly expressed in *C. elegans* and controls multiple facets of physiology, including aging, fat regulation, and development [39]–[43],[49],[53]. Previous studies have shown that DAF-2 regulates life-span by acting in neurons, while DAF-2 signaling in the muscles mediates intestinal fat levels [54]. To determine where DAF-2 is required to suppress sex-muscle seizures in response to food deprivation, we generated tissue-specific constructs using wild-type DAF-2 cDNA to rescue *daf-2(e1368) unc-103(0)* mutants (Table 3). We used the *lev-11* body-wall and sex-muscle promoter, the *tnt-4* pharyngeal muscle promoter, the pan-neuronal promoter of *aex-3*, and the intestinal *gtl-1* promoter. Only the *Plev-11:daf-2(+)* construct restored food-deprivation suppression of sex-muscle seizures, suggesting that DAF-2 acts in the muscles.

**Table 3:** Tissue-specific rescues of *daf-2(e1368)* in *daf-2(e1368) unc-103(0)* males.

Rescue construct^a^	Tissue Expression	% Protracted on Food	% Protracted on No Food
None	None	40 (212)	28 (138)
*rgEx178* [*Plev-11:daf-2(+)*]	Body-wall muscles, sex muscles	41 (37)	9^b^ (33)
*rgEx180* [*Paex-3:daf-2(+)*]	All neurons	14^c^ (21)	11 (27)
*rgEx179* [*Punc-1031E:daf-2(+)*]	Sex muscles, anal depressor, AIB, RIM, AIY,AVJ, ALA,AVH,NSM, I5	38 (26)	9^b^ (22)
*rgEx199* [*Punc-1031F:daf-2(+)*]	ALA, ADL, ASK, AVH, AVJ, AIN, AVA, ASJ, SMDD, SIA, ADE, AVD, I2, NSM, IL, IL2, OLL, URA, ASH, AVD, AUA,AUA, SIA, OLQ, RIV, URY, AIN, AIA, SPC, PCA, PCB, HSN, ray 1,2,3,4,6,9	46 (48)	29 (38)
*rgEx236* [*Pgtl-1:daf-2(+)*]	intestines	48 (33)	34 (41)
*rgEx237* [*Ptnt-4:daf-2(+)*]	pharyngeal muscles	41 (49)	38 (21)

a Background strain is daf-2(e1368) unc-103(0).

b Significantly different than the non-transgenic no-food control, Fisher's exact test.

c Significantly different than the non-transgenic food control, Fisher's exact test.

To determine if DAF-2 acts in the body-wall muscles or sex muscles to suppress spontaneous sex-muscle contraction, we expressed *daf-2(+)* in the sex muscles using the *unc-1031E* promoter. Promoter *Punc-103E* is expressed in approximately 7 pairs of neurons in the head, one pair of pharyngeal neurons and the spicule protractor muscles [16]. Interestingly, the sex-muscle specific *Punc-1031E:daf-2(+)* construct restored suppression of *unc-103(0)*-induced seizures. These results suggest that DAF-2 receptors expressed on the sex muscles respond to food-deprivation signals. It is therefore possible that the AWC olfactory neurons signal to the sex muscles directly or indirectly via regulating the release of insulin-like peptides.

In addition to acting in the sex muscles, we found that DAF-2/insulin-like receptor signaling can also function in neurons to regulate sex-muscle excitability. Surprisingly, the *Paex-3:daf-2(+)* construct significantly suppressed *unc-103-*induced seizures on the food condition, suggesting that increased DAF-2 signaling in the neurons can compensate for loss of UNC-103 function in the sex muscles (Table 3). However, this regulation is separate from *daf-2* regulation of sex-muscle excitability during food-deprived conditions, since there is no further decrease in seizure susceptibility in food-deprived males (Table 3). Although neuronal *daf-2* signaling may influence sex-muscle excitability on food, our results suggest that sex-muscle insulin-like receptor signaling reduces seizure susceptibility during food-deprived conditions.

**Figure 5:**
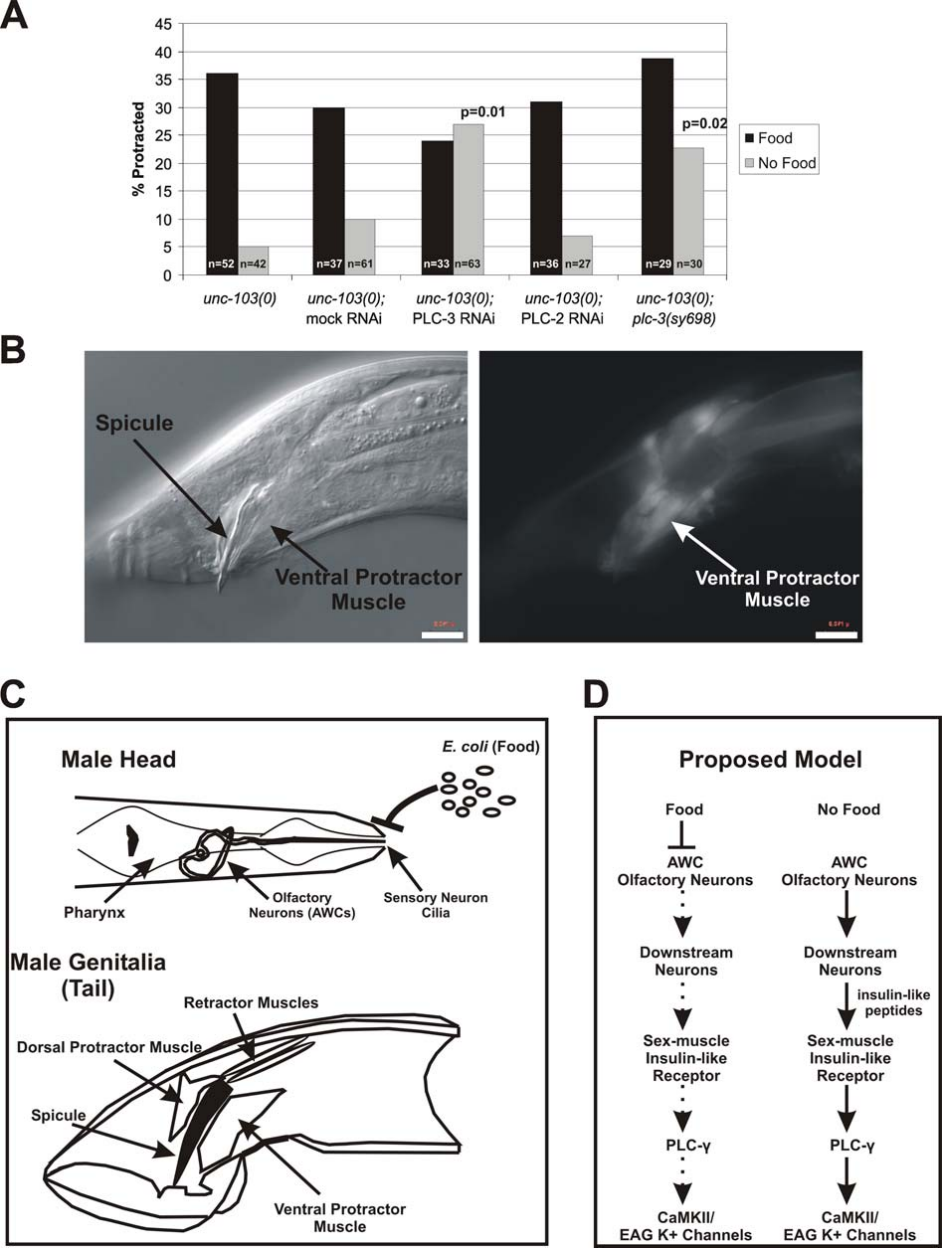
Phospholipase C-gamma (PLC-3) is required for food-deprivation suppression of *unc-103(0)*-induced muscle seizures. (A) Graph displaying the effects of PLC-3, PLC-2 RNAi, and *plc-3(sy698)* on *unc-103(0)*-seizure susceptibility on food and no-food conditions. p-value for PLC-3 RNAi relative to *unc-103(0)* Mock RNAi no-food control, p-value for *unc-103(0); plc-3(sy698)* relative to *unc-103(0)* no-food control (Fisher's exact test). (B) *Pplc-3:*YFP expression pattern in young adult male. The ventral protractors are labeled. Scale bar 6 µm. (C) Cartoon displaying the relevant structures in the head and male tail. (D) Proposed model for sensory regulation of sex-muscle excitability during food-deprivation. When food odors are present, AWC activity is attenuated. However, under food-deprived conditions, AWC activity is up-regulated and results in release of insulin-like peptides from downstream neurons. Insulin-like peptides activate insulin-like receptors on the male tail, resulting in activation of PLC-γ and CaMKII. CaMKII then reduces sex-muscle excitability through the activation of EAG K^+^ channels.

We have previously demonstrated that in starved males, CaMKII is responsible for suppressing sex-muscle seizures [1]. Since DAF-2 regulation of sex-muscle excitability does not require DAF-16, we looked for other targets of insulin-like receptor signaling that could affect CAMKII activity. Work by others has demonstrated that IGF-1 can activate CaMKII signaling pathways in human neuroblastoma and rat hippocampal neurons, and this occurs due to increased cystolic Ca^2+^ from the PLC-γ/IP_3_ activity [55]–[57]. To test if PLC-γ is required to suppress sex-muscle seizures under food-deprived conditions, we knocked-down the worm homologue of PLC-γ, PLC-3, via RNAi (Figure 5A). RNAi of PLC-3 significantly increased the percentage of males with spontaneous seizures on the no-food condition, whereas RNAi of another PLC-homologue, PLC-2 (PLC-β) had no significant change. To test this more rigorously, we constructed a *unc-103(0); plc-3(sy698)* [58] double mutant (Figure 5A). On plates with no *E. coli*, we found that 25% of *unc-103(0); plc-3(sy698)* double mutant males had spontaneous muscle seizures, compared to 5% of *unc-103(0)* males. These results are similar to the effects of the *daf-2(lf)* mutations, suggesting that *daf-2* may act through *plc-3* to regulate sex-muscle excitability.

In males, PLC-3 was previously reported to be expressed in the seminal vesicle valve cell and the vas deferens [59]. Using a larger upstream promoter region, we found that our 4.4 kb *plc-3* promoter construct also expressed in the ALA neuron (data not shown), the male retractor muscles, and the ventral protractor muscles (Figure 5B). By analogy with the mammalian cell-culture studies, these results suggest that under food-deprived conditions, DAF-2 signaling might stimulate PLC-3 activation of IP3 and the resulting increase in sex-muscle cystolic Ca^2+^ leads to activation of CaMKII, thereby reducing sex-muscle excitability.

We have previously reported that CaMKII activates the EAG-like K^+^ channel (EGL-2) in the sex muscles, suggesting that EGL-2 acts downstream of DAF-2 [1]. To test this directly, we generated a *daf-2(e1368) unc-103(0); egl-2(gf)* triple mutant and assayed the number of food-deprived males with seized muscles. We hypothesized that a constitutively active EGL-2 K^+^ channel should suppress *daf-2(lf)* mutants if the channel acts downstream of DAF-2. We found that this was indeed the case, as *daf-2(e1368) unc-103(0); egl-2(gf)* males were suppressed from 38% (n = 45) to 8% (n = 45) by food deprivation, which was significantly different than the 28% (n = 43) observed of food-deprived *daf-2(e1368) unc-103(0)* males (p = 0.03, Fisher's exact test).

## Discussion

Many animals encounter environments where food availability fluctuates and therefore they must endure periods of food limitation. As a result, animals have evolved adaptive physiological responses to ensure they survive until food becomes available again. The most well studied physiological responses to food availability are life-span and lipid homeostasis. In *C. elegans*, longevity and lipid homeostasis are regulated by chemosensory neurons, suggesting that perception of food in the environment, and not just intake of nutrients, can directly regulate physiology [60]–[62]. In mammals, while no direct link between sensory function and lifespan has been made, there is much evidence supporting the connection between chemosensory function and lipid metabolism [63]–[65]. In addition to promoting longevity and fat breakdown under food-stressed conditions, organisms alter their behavior by activating food seeking behaviors and suppressing other behaviors not involved in locating food. Specifically, in circuits not involved in food foraging, food deprivation likely activates signaling molecules that reduce cell-excitability. This natural physiological response could be a possible mechanism for why food deprivation has been observed to reduce seizure susceptibility in rodents [5]–[7],[66]. Understanding the pathways that regulate these adaptive physiological responses could lead to better health treatments for abnormal excitable cell output without the need of food deprivation.

We found that food availability regulates *C. elegans* sex-muscle excitability via two inputs, through sensation of food and ingestion of food. Loss of *unc-103*, which encodes an ERG-like voltage-gated K^+^ channel, causes about 40% of well-fed *C. elegans* males to suffer seizures of their sex muscles, whereas only ∼8% of food-deprived *unc-103* mutants display spontaneous seizures [1],[13]. We have previously identified that non-chemosensory neurons and organs, including the neuromuscular organ controlling food ingestion, the pharynx, can regulate sex-muscle excitability. Specifically, in tropomyosin/*lev-11* mutants, which phenocopy the effects of food deprivation on mating behavior, the pharyngeal neurosecretory motor neurons (NSMs) suppress *unc-103*-induced sex-muscle seizures [13]. However, it is still possible that lack of nourishment from food contributes to suppressed muscle seizures. In addition to affecting sensory neurons, food-deprivation induces cellular stress and/or damage. One way to measure this is to visualize the levels of autophagy, or the catabolism of cellular components, which increases during nutrient deprivation [67]. LGG-1 is the *C. elegans* ortholog of yeast Apg8/Aut7p and mammalian MAP-LC3, and can be used to mark preautophagosmal and autophagosomal membranes [68]–[71]. However, we found that cellular damage is not a likely mechanism, since the starvation period was not long enough to induce a significant increase in autophagy measured by a GFP-tagged LGG-1 (Figure S2A-B). Additionally, we used phalloidin to stain filamentous actin in fed and 15-hr food-deprived males and found no obvious defects in the muscle structure of food-deprived males (Figure S2C). Based on these results, we propose that reduction in male-mating efficiency and sex-muscle excitability by food deprivation is a regulated and reversible process, rather than the result of muscle damage or dysfunction.

In addition to food ingestion, food availability can regulate sex-muscle excitability via a chemosensory component. Interestingly, we found that food-deprivation reduction in *unc-103-*seizure susceptibility can be partially blocked by placing males in inedible food or by disrupting chemosensory function. We identified one pair of ciliated sensory neurons in the head, the AWCs, which can down-regulate sex-muscle excitability under food-deprived conditions. Ablation of these neurons in *unc-103(0)* males blocks the suppressing effects of food deprivation. Chalsani et al [38] demonstrated that the AWC neurons are activated upon odor removal, suggesting that these neurons are active when no food is being sensed in the environment. We found similar results while measuring AWC activity during our food-deprivation assays. Prior to food deprivation, males showed lower intracellular calcium levels that markedly increased during the food-deprivation interval and then decreased again when the males were placed on food. Thus, in males, AWC activity could also be initiating signals that down-regulate sex-muscle excitability.

In hermaphrodites, the AWC neurons mediate attraction to at least 5 attractive volatile odors, and also function to increase turning probability during local search behavior. Local search behavior allows worms that have been removed from food to explore limited areas by initiating a series of reversals and omega turns [24],[29],[72]. In males, the AWCs have recently been implicated in regulating male-sexual attraction behavior to hermaphrodite pheromones [32]. It is possible that in males, AWC-mediated attraction to hermaphrodites shares a similar mechanism to attractant chemotaxis in hermaphrodites. The AWCs likely regulate turning probability through glutamate-mediated synapses to the AIB and AIY interneurons, which can signal to the SMD and RIV head motor-neurons [38],[72]. However, in our studies, it is not exactly clear how the AWCs communicate to the male genitalia. Although there are clear sexually dimorphic differences in AWC function, both males and hermaphrodites appear to use these neurons to mediate attraction to an odorant source [24],[29],[32],[72]. Based on our AWC-G-CaMP experiment, we propose that, in males, AWC likely mediates attraction to food odorants as well as hermaphrodite pheromones.

We hypothesize that chemosensory neurons may coordinate muscle excitability with other physiological responses to food availability. Specifically, a common mechanism could be through regulating the release of neuroendocrine signals from downstream neurons that also regulate metabolism, such as insulin-like signals. Support for a link between chemosensory function and metabolism come from studies of the mammalian tubby gene, *Tub*, which is a highly conserved protein expressed in the central nervous system. Mice with loss-of-function mutations in *Tub* suffer from late onset obesity and have defects in ciliated neurons including retinal degeneration and hearing loss [73]–[75]. Similar to the mammalian studies, loss of *tub-1* function in *C. elegans* results in increased fat storage and defects in olfactory behavioral response mediated by the AWA and AWC olfactory neurons [62],[64],[73],[74]. Specifically, control of lipid homeostasis has been proposed to be dependent on the neuroendocrine function of a set of 15 ciliated neurons, including the AWC neurons [62]. Consistent with a role of chemosensory function in the control of fat breakdown in males, we found sensation of food suppresses mobilization of fat stores, independent of ingestion. Interestingly, when we reduced the male's ability to sense inedible *E. coli*, we saw a reduction in fat staining. This suggests that sensation of food by chemosensory neurons commonly block the effects of food deprivation on muscle excitability and fat mobilization. Additionally, we found insulin-like signaling, which is known to regulate lipid-homeostasis in hermaphrodites [18],[39], regulates sex-muscle excitability. Previous studies suggest that neuroendocrine regulation of fat storage is independent of DAF-2, since FOXO/DAF-16 mutants have no effect on *tub-1*-increases in fat storage [62]. Therefore, neuroendocrine regulation of sex-muscle excitability and fat storage may be via different signaling molecules, or commonly through a FOXO/DAF-16-independent insulin-like signaling mechanism.

Historically, insulin signaling has been proposed to be active under well-fed conditions and regulate the body's ability to handle food by internalizing glucose. In *C. elegans*, a similar paradigm has been proposed with DAF-2/insulin-like signaling. Food deprivation has generally been proposed to inhibit DAF-2 activity, which can lead to FOXO/DAF-16-mediated dauer formation and increased longevity [39]–[43]. In contrast to studies in longevity and dauer formation, our data indicates DAF-2 activity is required under food-deprived conditions to suppress excitable motor output. This difference could possibly be attributed to sexually dimorphic differences, since males have been reported to have a significantly longer lifespan than hermaphrodites [76]. Although DAF-2 is not required for increased male longevity compared to hermaphrodites, *daf-2(lf)* mutations still significantly increase male lifespan, suggesting that, similar to hermaphrodites, insulin-like signaling also regulates similar pathways in males. Therefore, we propose that the assumption of food deprivation generally leading to decreased DAF-2 activity in all tissues may not hold true in all biological contexts, and insulin-like signaling may be more complicated then originally thought under these conditions.

Interestingly, the requirement for active insulin-like signaling under food-deprived conditions has been investigated by groups studying other biological responses to food availability. For example, one characterized response to food deprivation, termed *F*asting-*I*nduced *R*edistribution of *E*sterase (FIRE), which results in the relocation of intestinal esterase activity from the cytoplasm to the nucleus during fasting, has been reported to require DAF-2 activity [77]. Loss-of-function mutations in *daf-2* suppress the FIRE response associated with food deprivation. Additionally, DAF-2 activity is required for salt chemotaxis learning, which allows worms that have previously been exposed to NaCl under starved conditions negatively chemotax away from a NaCl source [45]. Similarly, *C. elegans* also associate temperature with food deprived conditions by avoiding temperatures at which they were previously food deprived. This behavioral association of food and temperature requires the insulin-like ligand, *ins-1*, which appears to negatively regulate DAF-2 activity in this context [44]. The observation that *ins-1* is required to actively suppress DAF-2 activity under food-deprived conditions suggest that DAF-2 can be active under these conditions and must be inactivated for certain biological responses. Interestingly, *ins-1* is thought to act as a DAF-2 agonist for salt chemotaxis learning. Thus, the action of DAF-2 and insulin-like signals under different environmental conditions is likely more complex than a general activation or reduction in DAF-2 activity, and likely varies depending on the physiological response in question. This could possibly explain the requirement for so many insulin-like ligands [78] and the observations that certain DAF-2 responses such as reduction in sex-muscle output and salt-chemotaxis learning [45] do not require the FOXO/DAF-16 transcription factor.

Given our observations, we propose that sensory perception of food can regulate organismal physiology. In *C. elegans* males, we have demonstrated that suppression of mutant-induced seizures by food-deprivation requires chemosensory neurons and insulin-like signaling. Specifically, we propose that in low-food environments, AWC-olfactory neuron activity is up-regulated, leading to the release of insulin-like peptides by downstream neurons (Figure 5C-D). These peptides bind insulin-like receptors on the sex muscles and lead to the Ca^2+^ activation of CaMKII through PLC-γ. CaMKII then leads to reduced excitability by activating EAG K^+^ channels. This regulation of muscle excitability by chemosensory and insulin-like signaling may indicate a general mechanism used by organisms to regulate multiple behavioral and physiological responses to changing environmental conditions.

## Materials and Methods

### Strains

All strains contain *him-5(e1490)* (LGV) [79] and were maintained according to [80]. The following strains were used in this study: LGII: The *plc-3(sy698)* strain used in this study is PS5109 described previously in [58] and graciously provided by Cheryl Van Buskirk; LGIII: *daf-2(e1368)* [81], *daf-2(m41)* [49], *unc-103(n1213)* [23], and *pha-1(e2123)* [82]; LG IV: *osm-9(ky10)* [83]; LGV: *egl-2(n693)* [84]; LGX: *osm-5(p813)* [22].

### Behavioral Assays

Scoring of the spontaneous spicule-muscle seizure phenotype for the different feeding conditions was done as previously described [1]. Briefly, approximately 20–30 L4 males were separated and allowed to develop on one of three types of plates: NGM plates seeded with OP50, NGM plates without OP50, or aztreonam-LB/NGM plates seeded with pre-azetreonam-treated OP50 and scored 15–20 hours later for spontaneous spicule protraction. The protocol for aztreonam-NGM plates and aztreonam treatment is given below. Males that crawled up on the side of the plate and dried up were not scored. To minimize suicidal males, we used an 8M glycerol ring around the edge of the plates. For *daf-2(e1368)* lines, males were raised at 20°C until L2-L3 stage and then kept at 25°C for food-deprivation assays unless otherwise noted. For *daf-2(m41)* lines, males were raised at 15°C until L2-L3 stage and then raised and food-deprived at 25°C. For heat-shock rescue, males were heat-shocked at 33°C for 35 minutes prior to placing them on plates with or without bacteria. For all assays, males were picked from non-crowded and non-contaminated plates, and approximately 3–5 independent trials were performed.

For mating efficiency assays, we separated L4 males from hermaphodites and placed them on one of the three different feeding conditions, similar to the above assay. The next day, we then placed one male with one *pha-1(e2123)* hermaphrodite on 3.5cm NGM plate that was seeded with a .5cm diameter lawn of log-phase *E. coli* OP50 15hrs prior to the assay. Males were allowed to mate for 2hrs and then removed from the plate. Plates were then incubated at 25°C for 48hrs and then scored for the presence or absence of *pha-1(+)* growing F1 progeny. *pha-1(e2123)* is a temperature-sensitive mutation that allows growth at 15°C, but is lethal at 25°C.

### Aztreonam-Treated OP50 (Inedible Food)

OP50 cultures were grown to log growth in LB at 37°C with rigorous shaking. Once the cells reached log growth, aztreonam (Sigma) was added to a final concentration of 10 µg/ml, and the cells were grown an additional 3hrs with minimal shaking to minimize damage to the bacterial filaments. Cells were then spotted on LB plates containing NGM ingredients and 10 µg/mL aztreonam. Azteonam-treated bacteria and plates were used fresh (the same day), as plates older than 2 days had normal length bacteria, likely due to the drug being exhausted.

### Assay for the Effects of AWC Odorants on Spicule Protraction

To phenocopy the effect of the AWC ablation and *Podr-3:unc-103(gf)* experiments, we used odorants previously shown to reduce AWC activity. We used Sephadex G-50 beads (20–80 µm) (Sigma, www.sigmaaldrich.com) and acetone as a vehicle for the worms to chemotax to and contact. Specifically, we used a sterile M9 solution containing 10^−3^ butanone, 10^−4^ isoamyl alcohol, 10^−7^ 2,3-pentanedione, 10^−4^ benzaldehyde, and 30mg/ml Sephadex beads. 20 µl of this solution was spotted on a sterile NGM plate along with 1 µl of .4M sodium acetate. As a control, we spotted 20 µl of a M9 solution containing only 30mg/ml Sephadex beads along with 1 µl .4M sodium acetate on a sterile NGM plate. The sodium acetate ensured that the worms remained near the soaked beads, and showed no effects on spicule protraction mutants when used in isolation.

### Visualization of Fat Stores using Nile Red

Nile Red protocol was carried out as previously described [18]. Nile red was added to plates previously seeded with OP50, and larvae on these plates were synchronized by hypochlorite treatment. L4-stage males stained with Nile Red (www.mpbio.com) were then added to one of the three different feeding plates (OP50, aztreonam-treated OP50, or no OP50), then visualized for Nile Red staining 15 hours later using a fluorescent microscope (100X). To quantify the number of fat droplets, equal planes and the region immediately posterior to the pharynx were selected. Hermaphrodites were also visualized and showed a similar trend; however the data shown in the figure were obtained from males.

Analysis of Nile Red intensity changes were performed as described previously [18],[85]. Briefly, images were captured by focusing on the first two intestinal cells. Nile Red intensity density was then quantified using ImageJ software (U.S. National Institutes of Health, Bethesda, MD, USA; http://rsb.info.nih.gov/ij/). A region of interest was drawn around the first two intestinal cells and a 1.0 pixel Gaussian filter was used to identify fluorescent lipid droplets. The Analyze Particles function in ImageJ was used to count the number of lipid droplets, and generate a mask. The mask was then overlayed to the original ROI marking the first two intestinal cells, resulting in measurement of fluorescence in only in lipid droplets. Intensity Density (area×mean) of fluorescence specifically in lipid droplets is reported in Figure 2D.

### Pharmacology

Pharmacology was performed as previously described [1]. L4 males were separated from hermaphrodites and placed on either NGM plates seeded with OP50 or NGM plates with no OP50 for 15–20hrs, and then scored for drug sensitivity. Arecoline was obtained from Indofine Chemical Company (www.indofinechemical.com). Curve fits, EC90 and EC50 concentrations were generated using GraphPad Prism 4 software (GraphPad Software, Inc.).

### Plasmids

The details for the generation of plasmids and primers used in this study are listed in the Supporting Information (Text S1 and Table S1). pTG81 contains *pha-1(+)* from pBX1 and the *odr-3* promoter driving G-CaMP. pLR136 contains *pha-1(+)* and the *unc-103E* promoter driving G-CaMP. pTG71, pTG73, and pTG82 contain the *ocr-2*, *odr-3*, and *osm-12* promoters driving *unc-103(gf)*. pTG76, pTG78, and pLR89 contain YFP expressed from the *odr-3*, *ocr-2*, and *osm-12* promoters, respectively. pTG57, pTG58, pTG59, pTG60, pTG61, and pTG65 contain the *unc-103E*, *aex-3*, *gtl-1*, *lev-11*, *tnt-4*, and *unc-103F* promoters cloned in front of the *daf-2* cDNA. pDG9 and pTG6 contain the *lev-11* and *tnt-4* promoters driving GFP and have been described previously [13]. pTG92 contains a 4.4kb upstream region of PLC-3 plus the first 12 codons fused to YFP.

### Transgenics

To obtain *unc-103* transgenic lines, DNA was co-injected with pBX1 (100ng/µl) into *pha-1(e2131) unc-103(n1213)* hermaphrodites. The pBX1 plasmid contains *pha-1(+)* and was used to select for transformants [86]. For *unc-103(gf)* and YFP constructs, all constructs were injected at 25ng/µl.

For the *daf-2(+)* rescue constructs, DNA was co-injected with a GFP-marker into *daf-2(e1368) unc-103(n1213)* hermaphrodites. The *daf-2* rescue constructs and GFP markers were combined with pUC18 to bring the final DNA concentration to 200ng/µl and the injection mixtures for each rescue were: pTG60 (50ng/µl) and pDG9 (5ng/µl); pTG58 (50ng/µl) and pTG6 (20ng/µl); pTG57 (50ng/µl) and pDG9 (5ng/µl); pTG65 (50ng/µl) and pDG9 (5ng/µl); pTG59 (50ng/µl) and pTG6 (20ng/µl); pTG61 (50ng/µl) and pTG6 (20ng/µl). The GFP-markers were used to select for transgenic males and at least two independent lines were analyzed for each injection.

To visualize PLC-3 expression, pTG92 (50ng/µl) was injected along with pBX1(100ng/µl) into *pha-1* hermaphrodites. 8 independent transmitting lines were analyzed, one representative is shown in the Figure 5.

### Calcium Imaging with G-CaMP

pLR136 (*Punc-103E*:G-CaMP) was injected at 12ng/µl and pLR135 (*Punc-103E*:ds-Red) at 2ng/µl into *pha-1(e2123)*; *lite-1(e314)* hermaphrodites (The *lite-1(ce314)* strain was graciously provided by Dr. Ken Miller, Oklahoma Medical Research Foundation). Ten independent transmitting lines were obtained and two lines with low to moderate background G-CaMP fluorescence were further analyzed. L4 males were separated from hermaphrodites the night before and allowed to mature into adults overnight. Worms were placed on a pad containing 2% Noble Agar dissolved in sterile water, using a mouth pipette filled with S-Medium. The agar slide containing the worm was then placed on a cold block (∼1°C) for no longer than 15 seconds. This immobilized the male long enough to glue him down. A borosilicate capillary tube was pulled and broken, leaving an opening at the tip about .16mm in diameter. This tube was used to apply a small amount of glue (Nexaband S/C) to the male via mouth pipette near the dorsal posterior end of the animal. A drop of S-Medium was placed on top of the worm and then covered with a cover slip. Males were then observed using a 60x objective. The dorsal protractor muscles and anal depressor muscle were focused on and then the focus and exposure time were refined using fluorescence. Animals were then recorded for intensity changes using Image Pro Plus Version 6.2 software (Media Cybernetics). For drug exposure, a LED flash light was used to mark the beginning of drug application, and the drug was applied by pipetting 50 µl of drug the side of the coverslip. The drug then diffused across the pad and any intensity changes were recorded with Image Pro Plus. Fluorescence records were plotted as Δ*F*/*F*_0_, where *F*_0_ was the average baseline value of fluorescence before any drug stimulus. The sex muscles were marked as a region of interest using Image Pro Plus software, and the percent change in fluorescence intensity for this region was plotted for each time point relative to *F*_0_.

To measure G-CaMP intensity changes in the AWC neurons, pTG81 was injected into *pha-1(2123)*; *lite-1(ce314)* hermaphrodites at 35ng/µl. Six independent transmitting lines were obtained and one line with low background G-CaMP expression was used for analysis. Well-fed, virgin, adult males were washed with M9 buffer, and placed on a 1.5% agar pad containing no bacteria or sodium azide. Vacuum grease was then placed around the circumference of the pad followed by a cover-slip to seal in the moisture. Each male was labeled and intensity measurements were then recorded from the left AWC neuron at each time point of starvation at 100x. After starvation, males were removed from the agar plates, placed on NGM plates seeded with OP50 and allowed to re-feed for 15hrs. Males were then mounted again on the agar pads and the G-CaMP intensity in the left AWC neuron was recorded. At each time point, the fluorescence of the left AWC neuron and the background fluorescence of the isthmus of the pharynx were recorded. Intensity measurements were then plotted as the percent intensity change over time relative to the background fluorescence.

### RNAi of PLC-2 and PLC-3

To generate double-stranded RNA (dsRNA) for PLC-2, we PCR amplified a region of exon 9 using the primers FT7plc2 and RT7plc2 flanked by T7 promoter sequences. For PLC-3, we amplified exon 5 using the primers FT7plc3 and RT7plc3 flanked by T7 promoter sequences. The resulting PCR products were then used a template for *in vitro* transcription with a T7-MEGAshortscript kit from Ambion (Austin, TX). The final RNA concentration of each reaction was ∼2 µg/µl.

To perform RNAi, we placed L4 males in microfuge tubes containing 5 µl of dsRNA, and 15 µl of S-Medium. For controls with food, 2ul of concentrated OP50 was added as a food source along with 5 µl dsRNA and 13 µl S-medium. After 15hrs, males were removed from the microfuge tubes and scored for spontaneous protraction of their spicules.

### Laser Ablations

Laser ablations were performed as described previously [87]. The *odr-3* promoter driving YFP was used to identify the AWC neurons in *unc-103(n1213) pha-1(e2123)* males. Ablations were originally done at L4 stage, however no effect was seen, and the *Podr-3:YFP* construct still expressed in the AWC neurons even in the adult animal, suggesting that ablation in L4 males was too late. However, when ablations were done in L3 stage, YFP expression disappeared in the AWC neurons in adult males. To rule out any effects of the transgene or *pha-1(e2123)*, ablations of AWC were then done in L3-stage *unc-103(n1213)* males and allowed to recover at 15°C overnight. The next day, males were moved to assay plates (food, inedible food, or no food) at late L4 larval stage and then scored for spontaneous spicule protraction 15–20hrs later. Males that dried up and died on the side of the plates were not scored.

## Supporting Information

Figure S1 (A) Graph displaying chemotaxis index to isoamyl alcohol of males expressing chemosensory promoter-*unc-103(gf)* constructs. The * indicates a significant difference from non-transgenic controls (p-value < 0.05, Fisher's Exact Test). (B) Representative image of a male that was fed non-treated GFP-expressing *E. coli*. Arrow points to intact *E. coli* in the intestines (C) Representative image of a male fed aztreonam-treated GFP-expressing *E. coli*. Arrow points to inedible-aztreonam treated *E. coli*. (D) Graph displaying the number of cell nuclei observed in the gonad of L1-stage worms placed on one of the three feeding conditions for 15hrs (p-value Fisher's exact test). (E) Graph displaying the effect of *Podr-3:unc-103(gf)* and AWC ablation on wild-type male mating efficiency (p-value Fisher's exact test).
(3.93 MB TIF)

Figure S2 (A) Representative pictures of males expressing LGG-1 in 3 separate feeding conditions, Fed, Food Deprived for 15hrs, and Food Deprived for 3 days (scale bar 9 µm). (B) Graph displaying the number of LGG-1 puncta in males under four different conditions, Fed, Inedible Food 15hrs, No Food 15hrs, and starved for 3-days. For each condition, 10–20 males were analyzed. The * indicates a significant difference (p<0.001) then the other 3 feeding conditions. (C) Representative images of fed and food-deprived males stained with phalloidin, which stains filamentous actin (scale bar 20 µm). No differences were observed in muscle structure between the two conditions.
(3.30 MB TIF)

Table S1 Primers used in this study.
(0.04 MB DOC)

Text S1 Supporting materials and methods.
(0.06 MB DOC)
