# Supplementary figures and images for: Sensory Perception of Food and Insulin-Like Signals Influence Seizure Susceptibility
Source: PLoS Genet. 2008 Jul 4;4(7):e1000117. doi: 10.1371/journal.pgen.1000117 (PMC2432499; doi:10.1371/journal.pgen.1000117)

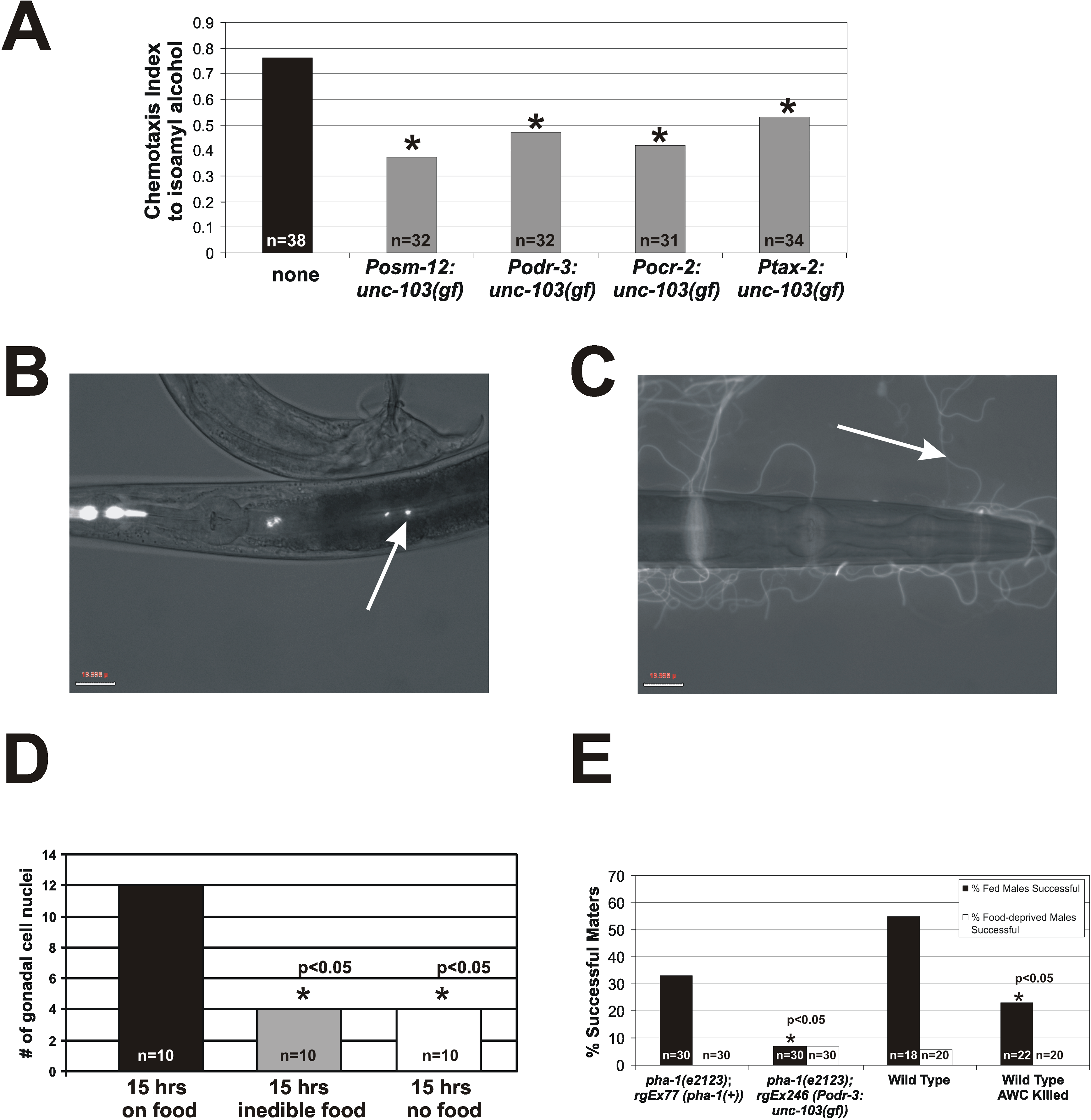

Supplement: Figure S1 — (A) Graph displaying chemotaxis index to isoamyl alcohol of males expressing chemosensory promoter-unc-103(gf) constructs. The * indicates a significant difference from non-transgenic controls (p-value < 0.05, Fisher's Exact Test). (B) Representative image of a male that was fed non-treated GFP-expressing E. coli. Arrow points to intact E. coli in the intestines (C) Representative image of a male fed aztreonam-treated GFP-expressing E. coli. Arrow points to inedible-aztreonam treated E. coli. (D) Graph displaying the number of cell nuclei observed in the gonad of L1-stage worms placed on one of the three feeding conditions for 15hrs (p-value Fisher's exact test). (E) Graph displaying the effect of Podr-3:unc-103(gf) and AWC ablation on wild-type male mating efficiency (p-value Fisher's exact test). (3.93 MB TIF) [file pgen.1000117.s001.tif]

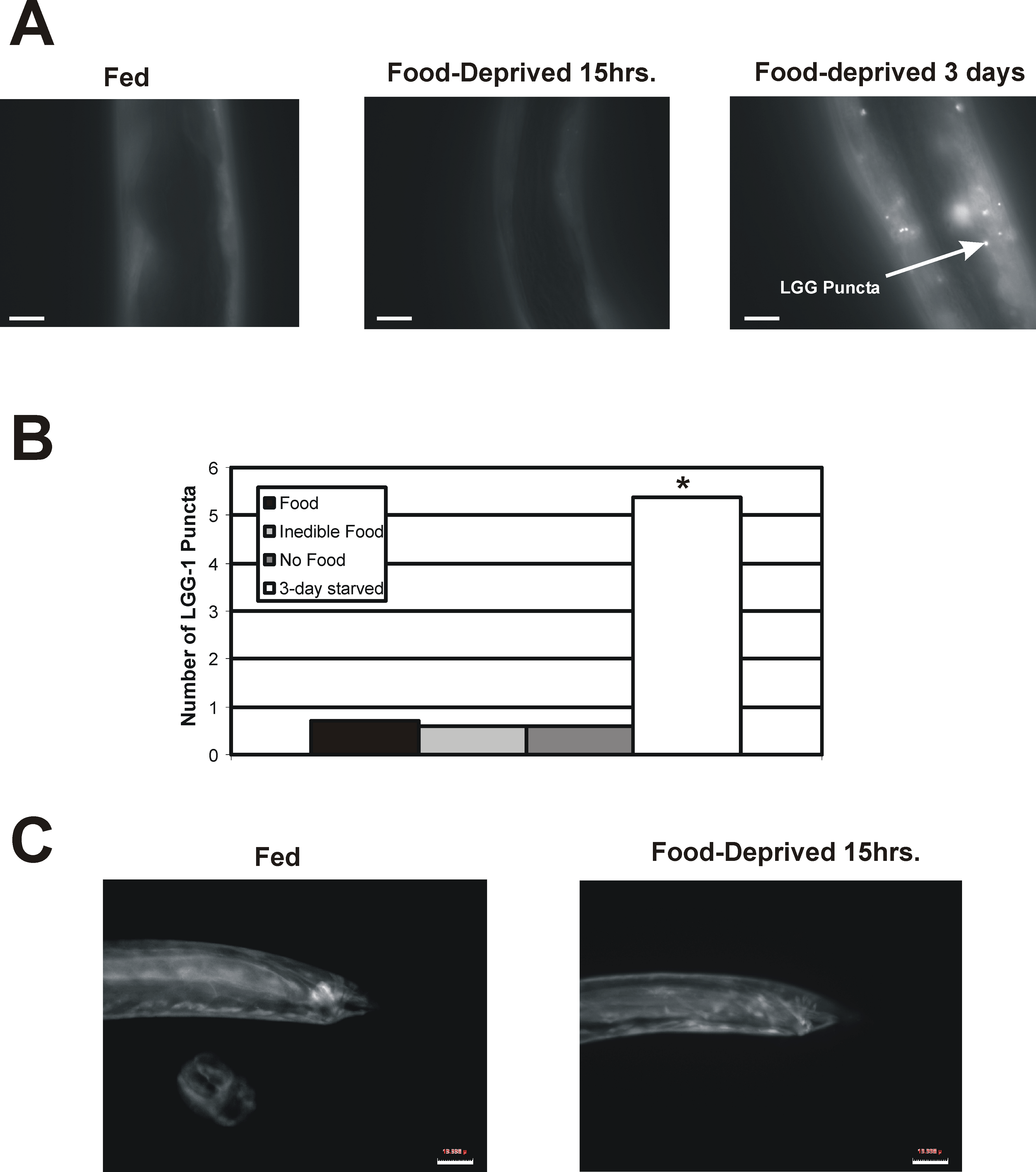

Supplement: Figure S2 — (A) Representative pictures of males expressing LGG-1 in 3 separate feeding conditions, Fed, Food Deprived for 15hrs, and Food Deprived for 3 days (scale bar 9 µm). (B) Graph displaying the number of LGG-1 puncta in males under four different conditions, Fed, Inedible Food 15hrs, No Food 15hrs, and starved for 3-days. For each condition, 10–20 males were analyzed. The * indicates a significant difference (p<0.001) then the other 3 feeding conditions. (C) Representative images of fed and food-deprived males stained with phalloidin, which stains filamentous actin (scale bar 20 µm). No differences were observed in muscle structure between the two conditions. (3.30 MB TIF) [file pgen.1000117.s002.tif]
