# Supplementary material for: Sensory Perception of Food and Insulin-Like Signals Influence Seizure Susceptibility
Source: PLoS Genet. 2008 Jul 4;4(7):e1000117. doi: 10.1371/journal.pgen.1000117 (PMC2432499; doi:10.1371/journal.pgen.1000117)
Supplement: Table S1 — Primers used in this study. (0.04 MB DOC) [file pgen.1000117.s003.doc]

**Supporting Table**

Table S1: Primers used in this study

| **Primer Name** | **Sequence** |
| --- | --- |
| Attb1daf2 | GGGGACAAGTTTGTACAAAAAAGCAGGCTTCATGAATATTGTCAGATGTCG |
| Attb2daf2 | GGGGACCACTTTGTACAAGAAAGCTGGGTCTCAGACAAGTGGATGATGCTC |
| AttB1Pocr2 | GGGGACAAGTTTGTACAAAAAAGCAGGCTTCGAGTACTTCGACTACTGGATGTAAG |
| AttB2Pocr2 | GGGGACCACTTTGTACAAGAAAGCTGGGTCCTTAATGATGTGATGTACTCTACTG |
| AttB1Podr3 | GGGGACAAGTTTGTACAAAAAAGCAGGCTTCGCAAGACCAATTAGCAACTCGCTCC |
| AttB2Podr3 | GGGGACCACTTTGTACAAGAAAGCTGGGTCCTATGAGTAATTGATTTGAAATATCG |
| Attb1PO12 | GGGGACAAGTTTGTACAAAAAAGCAGGCTGTACTACACGTGGACAAGCG |
| RAttb2PO12 | GGGGACCACTTTGTACAAGAAAGCTGGGTTTTTTACAGTGGAAGCTGAG |
| Attb1Ptax2 | GGGGACAAGTTTGTACAAAAAAGCAGGCTTCGATCGGTTGACAATCAGTAGC |
| Attb2Ptax2 | ACCACTTTGTACAAGAAAGCTGGGTCATCGGAAAACTCCGGTTTTTCTGAC |
| ATTP1 | GACGTTGTAA AACGACGGCC AGTCTTAAGC |
| ATTP2 | GGCCAGAGCT GCCAGGAAAC AGCTATGACC |
| FT7plc2 | TTAATACGACTCACTATAGGGAGAGCAACCAACTGGTCTACGC |
| RT7plc2 | TTAATACGACTCACTATAGGGAGACCACAACCTCCATTTCTCTC |
| FT7plc3 | TTAATACGACTCACTATAGGGAGATAGATTGCTGGGATGGAC |
| RT7plc3 | TTAATACGACTCACTATAGGGAGAACTCATCAGTTTTCACCAC |
| Attb1Pplc3 | GGGGACAAGTTTGTACAAAAAAGCAGGCTTCGGTCCTCATCAGTACCCCACCACCAG |
| Attb2Pplc3 | GGGGACCACTTTGTACAAGAAAGCTGGGTCAGACGACGATGATGGGCCAAGTGAGC |
| HIIIYFP | GGGAAGCTTATGAGTAAAGGAGAAGAACTTTTCACTGGA |
| NRLGGYFP | GTTGTTCTCCTCCTTGTAAGCCCACTTCATTTTGTATAGTTCATCCATGCCATGTGTAAT |
| NATTB2LGG | ATTACACATGGCATGGATGAACTATACAAAATGAAGTGGGCTTACAAGGAGGAGAACAAC |
| XBALGG | GGGTCTAGAGAAGTGATAGTCTATGACTAGAAAGCGAGAC |
| attb1hsp | ggggacaagtttgtacaaaaaagcaggcttaagcttgcatgcctgcagg |
| attb2hsp | ggggaccactttgtacaagaaagctgggtgctagccaagggtcctcct |
