# Supplementary material for: Sensory Perception of Food and Insulin-Like Signals Influence Seizure Susceptibility
Source: PLoS Genet. 2008 Jul 4;4(7):e1000117. doi: 10.1371/journal.pgen.1000117 (PMC2432499; doi:10.1371/journal.pgen.1000117)
Supplement: Text S1 — Supporting materials and methods. (0.06 MB DOC) [file pgen.1000117.s004.doc]

**Text S1**

Supporting Materials and Methods

*Plasmids.* Primers used in this study are given in Table S1. To place promoters upstream of G-CaMP, the MCS, synthetic intron and *unc-54* 3’ UTR was cut out of pPD49.26 (A. Fire) using ApaI (blunted with T4 DNA polymerase) and HindIII. This fragment was then cloned into the AvaI (blunted) and HindIII sites of pBR322 to make pTG24. A region containing G-CaMP was cut out of the vector pN1-G-CaMP1.3 [1] using NotI (blunted) and BglII, and cloned into the SmaI-BamHI sites of pTG24 to make pTG29. pBX1 was cut with XhoI (blunted) and XbaI, and the 7.3kb fragment that contains the *pha-1* promoter and gene was cloned into the EcoRI (blunted) and XbaI sites of pTG29 to generate pLR133. The Gateway Cassette C.1 (Invitrogen) was then blunt-end cloned into the XbaI site of pLR133 to generate pLR134. This allows various promoters to be recombined upstream of G-CaMP. To drive expression of G-CaMP in the sex-muscles, the plasmid pLR21[2], which contains the *unc-1031E* promoter, was recombined with pLR134 using LR clonase (Invitrogen) to generate pLR136. To drive G-CaMP in *odr-3* expressing neurons, pTG70 (described below) was recombined with pLR134 LR clonase to generate pTG81.

Plasmids containing *unc-103* genomic DNA were created as previously described [2]. pLR62 contains *unc-103* genomic DNA and was used as template to generate the A331T mutation that exists in *unc-103(e1597gf)* using single-site mutagenesis to generate pLR73. To generate chemosensory promoters driving YFP and *unc-103(gf)*, we amplified promoter sequences flanked with attB sites and recombined each into the Gateway entry vector, pDG15 [2] using BP clonase (Invitrogen). pTG68 contains 3.8kb of *ocr-2* upstream sequences, pTG70 contains 2.7kb of *odr-3* upstream sequences, pLR88 contains 1.2kb of *osm-12* upstream sequences, and pTG87 contains 1.9kb of *tax-2* upstream sequences. These promoter-containing plasmids were then recombined using LR clonase into pGW322YFP or pLR73, which contain attR recombination sites in front of YFP and *unc-103(gf)*, respectively.

To generate *daf-2(+)* rescue constructs, the *daf-2* cDNA was amplified from a cDNA library using primers flanked with attB sites (Invitrogen). A 1.1kb region from pDONR221 containing an *att*P1 site, *ccdB* gene, chloramphenicol resistance gene, and *att*P2 site (Invitrogen) was PCR amplified (primers ATTP1 and ATTP2) and cloned into the SmaI site of pTG24 to create pTG38. The *daf-2* cDNA was then recombined into pTG38 to make pTG39. The *daf-2* cDNA was sequenced in pTG39 and 4 mutations were fixed using site-directed mutagenesis, to make pTG53. To place promoters in front of *daf-2*, the 1.1kb region of pDONR221 was cloned into the XbaI site of pTG53 to generate pTG54. Promoter regions for *unc-103E*, *aex-3*, *gtl-1*, *lev-11*, *tnt-4*, and *unc-103F* were amplified using the primers listed in Table S1 and recombined into pTG54 using BP clonase to generate pTG57, pTG58, pTG59, pTG60, pTG61, pTG65, respectively. For the heat-shock rescue construct, the *hsp-16* promoter was amplified with primers listed in Table S1 and then recombined into pTG54 to generate pTG56.

To generate a PLC-3 promoter-YFP reporter, we amplified a region containing a 4.4kb upstream region of PLC-3 plus the first 12 codons with primers flanked with attb sequences. This fragment was then recombined into pDG15 using BP clonase to generate pTG91. pTG91 was then recombined with pGW322YFP using LR clonase to generate pTG92.

To generate a GFP-tagged LGG-1, we first used the primers HIIIYFP and NRLGGYFP to amplify an 800bp fragment of pGW322YFP which contains YFP. We then used the primers NATTB2LGG and XBALGG to amplify a 2.3kb region of N2 DNA that includes the *lgg-1* gene and 3’UTR. We then used PCR sewing to combine the 2.3kb and 800bp regions using the HIIYFP and XBALGG primers. This fragment was then cut with XbaI and HindIII and cloned into the XbaI and HindIII sites of pTG24 to generate pLR139. We then blunt-end cloned the Gateway Cassette C.1 into the HindIII site of pLR139 to generate pLR141. This allows for various promoters to be recombined in front of LGG-1::YFP. pLR22 contains the *lev-11* promoter cloned into pDG15 [2, 5]. pLR141 and pLR22 were then recombined using LR clonase to generate pTG90 (*Plev-11:LGG-1::YFP*).

*Assay for chemosensory defects of promoter:unc-103(gf) constructs.* Young adult males were assayed for chemotaxis to isoamyl alcohol using a variation of the protocol previously described [3, 4]. Briefly, 1µl of isoamyl alcohol and 1µl of 1M sodium azide was placed on one side of a NGM plate while 1µl of water and 1µl of 1M sodium azide was placed at the other end of a NGM plate. Chemotaxis index was determined based on the number of animals at the attractant and transgenic animals were compared to wild-type controls.

*LGG-1 analysis.* Autophagy is induced when cells cannot gain enough nutrients from the extracellular environment and results intracellular protein breakdown by the lysosome. Under these conditions, LGG-1 expression is punctate, whereas under well nourished conditions, the expression of LGG-1 is diffuse [5-7]. To visulalize autophagy in the head and body-wall muscles, we injected pTG90 (*Plev-11:LGG-1::YFP)* at 10ng/µl along with pBX1 (100ng/µl) and pUC18 (90ng/µl) into *pha-1* hermaphrodites. 5 independent transgenic lines were obtained and analyzed. To quantify the effects of food deprivation on autophagy levels, we visualized the dorsal and ventral head muscles for punctate expression in fed and 15 hour or 3 day food-deprived males. Males were picked as L4, and then placed on one of three conditions, NGM plates with *E. coli*, with aztreonam-treated *E. coli*, or without *E. coli*. Males were then visualized using fluorescence microscopy. LGG-1 puncta were not visualized after our standard 15-hour deprivation assay. However, puncta began emerging as starved animals grew older (3 days).

*Feeding GFP-expressing Bacteria*. To determine the edibility of aztreonam-treated *E. coli*, we used a *CV2* strain of *E. coli* which constitutively expresses a GFP-containing plasmid (graciously provided by Gus Wright and Michael Manson, Texas A&M University). To test the edibility of aztreonam-treated *E. coli*, we treated this strain with aztreonam as described earlier and placed males on both treated and un-treated GFP-expressing bacteria. We found that we could visualize GFP-bacteria in the isthmus and grinder of the pharynx and in the intestines of males placed on the untreated bacteria. However, we never saw GFP bacteria in the intestines of aztreonam-treated GFP-bacteria, though in about 50% of the animals, what appeared to be a long-chain of bacteria was seen trapped in the isthmus of the pharynx.

*Phalloidin Staining*. L4 males were grown overnight on NGM plates with or without *E. coli* OP50. The next day, we removed worms from the plates and washed 2 times with S-basal to remove bacteria. Worms were then frozen in liquid nitrogen using an eppendorf tube and then lyophilized in a speedvac. 3-4 drops of cold acetone were then added for 5 minutes, and then dried off using a speedvac. 2U of conjugated phalloidin was then added to an eppendorf tube and the methanol was removed using a spedvac. The phalloidin was then resuspended in a 1ml solution containing 250µl .8M Na phosphate, 1µl 1M MgCl2, 4µl 1% SDS, and 743µl distilled water. This solution was then added to the dried worms and allowed to stain for about 1hr. Worms were then washed 2x with 1ml PBBT (PBS+ .5% BSA + .5% Tween-20). Worms were then mounted on an agar slide and viewed using a fluorescent microscope.

**References**

1. Nakai, J., Ohkura, M., and Imoto, K. (2001). A high signal-to-noise Ca(2+) probe composed of a single green fluorescent protein. Nat Biotechnol *19*, 137-141.

2. Reiner, D.J., Weinshenker, D., Tian, H., Thomas, J.H., Nishiwaki, K., Miwa, J., Gruninger, T., Leboeuf, B., and Garcia, L.R. (2006). Behavioral genetics of *Caenorhabditis elegans* *unc-103*-encoded erg-like K(+) channel. J Neurogenet *20*, 41-66.

3. Bargmann, C.I., Hartwieg, E., and Horvitz, H.R. (1993). Odorant-selective genes and neurons mediate olfaction in *C. elegans*. Cell *74*, 515-527.

4. Colbert, H.A., Smith, T.L., and Bargmann, C.I. (1997). OSM-9, a novel protein with structural similarity to channels, is required for olfaction, mechanosensation, and olfactory adaptation in *Caenorhabditis elegans*. J Neurosci *17*, 8259-8269.

5. Kabeya, Y., Mizushima, N., Ueno, T., Yamamoto, A., Kirisako, T., Noda, T., Kominami, E., Ohsumi, Y., and Yoshimori, T. (2000). LC3, a mammalian homologue of yeast Apg8p, is localized in autophagosome membranes after processing. Embo J *19*, 5720-5728.

6. Suzuki, K., Kirisako, T., Kamada, Y., Mizushima, N., Noda, T., and Ohsumi, Y. (2001). The pre-autophagosomal structure organized by concerted functions of APG genes is essential for autophagosome formation. Embo J *20*, 5971-5981.

7. Kim, J., Huang, W.P., Stromhaug, P.E., and Klionsky, D.J. (2002). Convergence of multiple autophagy and cytoplasm to vacuole targeting components to a perivacuolar membrane compartment prior to de novo vesicle formation. J Biol Chem *277*, 763-773.
